# Visualizing risk factors of dementia from scholarly literature using knowledge maps and next-generation data models

**DOI:** 10.1186/s42492-021-00085-x

**Published:** 2021-06-24

**Authors:** Kiran Fahd, Sitalakshmi Venkatraman

**Affiliations:** Department of Information Technology, Melbourne Polytechnic, 3181 Prahran, VIC Australia

**Keywords:** Big data, Data visualization, Knowledge maps, Dementia, Non-relational database, Graph database, Neo4j, Semantic visualization

## Abstract

Scholarly communication of knowledge is predominantly document-based in digital repositories, and researchers find it tedious to automatically capture and process the semantics among related articles. Despite the present digital era of big data, there is a lack of visual representations of the knowledge present in scholarly articles, and a time-saving approach for a literature search and visual navigation is warranted. The majority of knowledge display tools cannot cope with current big data trends and pose limitations in meeting the requirements of automatic knowledge representation, storage, and dynamic visualization. To address this limitation, the main aim of this paper is to model the visualization of unstructured data and explore the feasibility of achieving visual navigation for researchers to gain insight into the knowledge hidden in scientific articles of digital repositories. Contemporary topics of research and practice, including modifiable risk factors leading to a dramatic increase in Alzheimer’s disease and other forms of dementia, warrant deeper insight into the evidence-based knowledge available in the literature. The goal is to provide researchers with a visual-based easy traversal through a digital repository of research articles. This paper takes the first step in proposing a novel integrated model using knowledge maps and next-generation graph datastores to achieve a semantic visualization with domain-specific knowledge, such as dementia risk factors. The model facilitates a deep conceptual understanding of the literature by automatically establishing visual relationships among the extracted knowledge from the big data resources of research articles. It also serves as an automated tool for a visual navigation through the knowledge repository for faster identification of dementia risk factors reported in scholarly articles. Further, it facilitates a semantic visualization and domain-specific knowledge discovery from a large digital repository and their associations. In this study, the implementation of the proposed model in the Neo4j graph data repository, along with the results achieved, is presented as a proof of concept. Using scholarly research articles on dementia risk factors as a case study, automatic knowledge extraction, storage, intelligent search, and visual navigation are illustrated. The implementation of contextual knowledge and its relationship for a visual exploration by researchers show promising results in the knowledge discovery of dementia risk factors. Overall, this study demonstrates the significance of a semantic visualization with the effective use of knowledge maps and paves the way for extending visual modeling capabilities in the future.

## Introduction

In this digital era, massive datasets available in different forms, termed big data, grow rapidly with their complex structures derived from disparate sources such as the Internet, mobile devices, software/network logs, and social media. Advancements in information and communication technology (ICT) have led to the adoption of a flexible data management system that has the capability to store and process diverse, complex, and massive datasets [1]. Future ICT developments will focus on realizing the value of big data to derive higher positive impacts. Hence, the research focus relating to big data is not simply about storing and retrieving data, it is also about analyzing, documenting, and systematically extracting information for specialized purposes in a user-friendly and adaptive manner. Furthermore, the adoption of visualization techniques such as knowledge maps and graphs can deliver incredible benefits, particularly for a performance enhancement in every application domain.

A semantic visualization is a powerful data presentation technique that not only displays hidden knowledge in big data it also demonstrates the patterns of knowledge flow. Such visualization plays an important role in understanding, interpreting, and analyzing datasets based on the patterns of massively scaled and complex knowledge flows. There are various options available to visually present the data and knowledge, such as mind, organizational, concept, story, and graph-based maps. A particular visualization technique called a knowledge map is widely used to visually represent data in the database, in the form of nodes and edges that derive roots from graph theory.

According to ref. [2], there is no formal definition of a knowledge map. A knowledge map is represented by a labeled schematic drawing of the data source structure with nodes and relationships between the nodes connecting them, allowing for navigation through an endless chain. It provides information regarding the knowledge assets stored in big data in the form of a graph with vertices denoting the nodes of data points and arcs representing relationships between the nodes as association rules. As an advantage of a data visualization using a knowledge map, it can be used for both knowledge modeling and analytical computations of big data. This paper provides insight into the significance of the visual representation of data in the form of knowledge maps to effectively analyze and utilize the data and related information. Knowledge maps, also known as knowledge graphs, create machine understandable knowledge by linking nodes of related information, and can be implemented using graph databases to store the information captured as big data.

In this research, massive information collected in scholarly articles reporting the research findings is considered. Such studies are predominantly stored and archived as documents and are only recently being communicated in digital form. Their content can be found in the form of text, figures, tables, images, mathematical formulae, and many other unstructured formats, leading to challenges in knowledge retrieval from such big data repositories. Despite the rapid advancements in ICT, automatic knowledge derivation from scholarly articles found in the literature is still in its infancy owing to the difficulty in processing and analyzing such complex unstructured data. This is a drawback, particularly in the healthcare domain, which requires deep insight into the scholarly literature for quick evidence-based decision-making, such as the correct diagnosis of a disease by deriving risk factors from these unstructured repositories and their association rules. For instance, with rapid changes in the environment, there have been growing modifications in the risk factors of dementia reported in the literature [3]. However, the predominantly adopted keyword-based search and information retrieval from scholarly articles do not meet the required timely and accurate derivation of domain-specific knowledge by communities of practice in this digital age. Automated visualization techniques are suggested because seeking deep insights into this domain knowledge is challenging and time-consuming. In this context, it was observed that the recent developments in the database management of modern ICT have not been exploited to the full extent in terms of incorporating semantic visualization of big data that can facilitate timely and effective decision-making. These gaps form the key motivation of this research in proposing automated techniques for semantic visualization with a useful practical application for knowledge discovery for dementia risk factors.

In this paper, an innovative model is proposed for integrating non-relational database techniques of big data and knowledge map techniques to achieve a semantic visualization of knowledge derived from scholarly articles for practical applications. A non-relational graph database tool such as Neo4j is used to represent the relevant big data from scholarly articles and to develop the semantic visualization in the form of a knowledge map. The effectiveness of the proposed model is demonstrated by applying it to scholarly articles related to dementia risk factors and their association rules for enhancing the associated decisions in diagnosis as a case study.

Overall, the main purpose of this study is to identify a better automatic approach for retrieving, storing, and visualizing knowledge entities and their hidden relationships found in research articles to assist researchers in a deeper understanding of the literature. The present research study aims to achieve this through three key contributions: (1) An automated literature-based knowledge discovery is proposed with semantic visualization using knowledge maps as the first of its kind; (2) A novel graph-based model is used that represents important information from scholarly articles to facilitate researchers with an easy visual navigation through the literature that incorporates novel visual querying of non-relational big data graph repositories; (3) A prototype of the model combining big data and knowledge map techniques is implemented for evidence-based semantic reasoning of dementia risk factors using text data mining, meaningful entities and their relationships, graph datastores, and graph exploration applications for knowledge discovery with visual queries.

The remainder of this paper is organized as follows. First, a review of related studies is provided as the research background and to illustrate the need for the proposed model for semantic visualization. Next, the key features of using the knowledge map technique for semantic visualization are highlighted, thereby justifying its academic and practical value applicable to the present research context. A novel model is then proposed that integrates the big data graph store technique to extract relevant information from scholarly articles and a knowledge mapping technique for generating semantic visualization. Following this, the application of the proposed model and its implementation are demonstrated as a proof-of-concept in the field of dementia as a case study illustrating visual knowledge discovery. Three case scenarios are provided to demonstrate and verify its usefulness for researchers to visually query the graph data ore without having to write the commands. Finally, some concluding remarks and future research directions are provided.

## Related studies and the need for the present research

In the academic world and communities of practice, scholarly articles in the literature provide domain-specific knowledge, and their importance is weighed based on the credibility of the source of information, authors, and many other factors. It is common practice to extract metadata from research articles to provide relevant data in a structured manner [4]. The major findings from the literature review show that some of the standard information extracted from the research articles includes the title, author, year of publication, publisher, and editors, which are recorded in a database to create an open library publication framework. Such techniques assist in adding information about the research articles to a digital library repository. However, the primary focus has been to merely provide a representation of metadata to link and manage them for users to operate with a digital infrastructure. The main purpose is to facilitate a data exchange and communication among publishers, infrastructures, datasets, and different communities of interest. Examples of such research work include the Scholix project linking infrastructures such as Crossref and DataCite [5] and the research graph that links researchers and publications [6]. Another research object project relates specific entities, such as the research investigation artifacts from the abstracts of the articles [7]. A recent study was aimed at enhancing document-based scholarly communication by integrating crowdsourcing and automated techniques using semantic representations of communicated scholarly knowledge [8]. However, such existing studies have reported major limitations for a quick and effective knowledge discovery. They do not provide a flexible semantic visualization of the data from domain-specific scholarly articles with the aim of uncovering interesting patterns required for knowledge discovery that can assist in evidence-based decision-making. For example, in the case of health diagnosis with decision-making associated with dementia risk factors, a significant amount of research on the growing changes in attributing factors owing to environmental and societal impacts has been reported in scholarly articles [3, 9–11]. An automated visual representation of these changing attributing factors and their relationships from the literature would benefit researchers, practitioners, and communities of interest toward enhancing their productivity in decision-making.

Several studies have focused on the visualization of data in various forms and types, demonstrating how such data can improve understanding and reduce knowledge gaps owing to its graphical or visual representation for increasing the use of evidence-based research [5, 7]. With a massive number of scholarly articles published daily in healthcare domains, researchers and the community of practice prefer a faster analysis and decision-making using knowledge visualization [12, 13]. Although a visualization can uncover valuable patterns from big data, assist in sharing knowledge, and derive judgments for quick decision-making, there are challenges because such visualization generally represents snapshots raising the reliability of the findings to cope with dynamic changes [14, 15]. The impact of knowledge visualization and its acceptance depends greatly on the selection of visual representation techniques that enhance a semantic or cognitive understanding. Over the past few years, different approaches, techniques, and tools have been developed for semantic visual representations. The most popular options for semantic visualization are mind, concept, ontology, and knowledge (asset) maps. Therefore, the selection of a visualization technique that can effectively present domain data and association rules is critical.

A mind map represents the data as a drawing that shows the link between different elements of information and can assist in theory-refining for an individual. It consists of a network of concepts with the main topic placed in the center that connects other relevant ideas emanating from the central topic [16]. Mind maps avoid linear thinking and graphically represent the information to facilitate brainstorming about an idea or problem solving [17, 18]. However, these features do not fit well for visually associating important attributes forming big data that require a structured representation from large scholarly articles.

Concept maps were originally defined to represent database schemas at the conceptual level in a structured manner. However, over time, concept maps have evolved for use in a wide range of applications. The concept map structure is composed of two types of nodes: concept and link nodes. A concept node represents the entities, and a link node represents the relationships among the entities. A concept map is used to generate new information about a particular domain in a more formal and structured hierarchy than a mind map [18–20]. In a concept map, the primary, secondary, and tertiary ideas are represented in layers to form a conceptual framework that can be used for planning or evaluation [21]. Although concept maps are more structured than mind maps, their purpose is to generate new concepts, and they lack the features required to aid in domain-specific decision-making.

Ontology maps represent the connections between shared concepts across heterogeneous domains, providing explicit specification of a conceptualization [22]. The connection links present the technical vocabulary of the domain [23]. Semantic web and machine learning techniques are applications of an ontology map and have the potential for achieving better structures, such as organizational maps, and are represented as story maps for mapping narratives. An organizational mapping of an ontology visually displays information about an internal hierarchy, roles, and responsibilities within an organization divided into departments and operational units. By contrast, a story map graphically organizes the plot, controlling idea, setting, and characters of a story to clearly identify each element of the story [24]. A key benefit of using an ontology map is to establish communication among different knowledge systems and gather more accurate knowledge [16]. In the present research context of knowledge discovery from scholarly articles, an ontology map could be used to enable searches in different publications from different domains with a shared ontology model [25]. It has been reported that different ontology mapping models have faced limitations in transferring domain-specific knowledge from various sources to an effective representation; however, such issues were considered with better mapping techniques between relational databases and ontologies [26–28]. Some studies have considered using extremely large databases and natural language querying through ontologies to enhance interfaces for search requests and information retrieval that are closer to user requirements [29, 30]. However, such studies lack the visual representation of unstructured data in the context of big data collection of scholarly articles.

In ref. [31], a comparison among various ontology-based approaches for information retrieval including database-to-ontology and ontology-to-database mappings was conducted. A recent study examined the use of ontologies and semantic technologies to reduce the linguistic and conceptual gaps between user queries and data sources [32]. However, from such studies, it was suggested that future work toward the success of ontologies relies on integrating advanced techniques for language processing and an expert assessment for a better integrated automation. In addition, the lack of studies using an ontology map with non-relational databases in a big data environment is currently a drawback.

The majority of researchers in this field have reported the advantages of using a knowledge map as an effective technique for storage and intelligent traversal of knowledge [19, 20, 33]. According to ref. [34], semantic visualization in the form of an ontology-based knowledge map helps identify the connections among various existing domain knowledge, which improves evidence-based decision-making to address key domain-specific issues. In addition, the benefits of knowledge maps, such as identifying existing knowledge over time and identifying the flow of existing knowledge in an organization have been reported in multiple studies [18–20, 35]. Many-to-many complex relationships among scholarly articles and their underlying data were recently preserved as a knowledge map in the RMap Project [12], and the integration of bibliographic metadata in a knowledge graph was also proposed [13]. However, little work has been done to automatically extract and organize the knowledge from research articles as a formal semantic representation and visual integration using a big data repository. The proposed model fills this gap and aims to facilitate researchers to seek and relate existing knowledge using an interactive semantic knowledge map and integrated non-relational data storage for the visual navigation of a graph-based big data repository.

## Key features of knowledge maps for semantic visualization of scholarly articles

According to ref. [36], good data visualization has a higher impact than written text-based reports. It is critical to understand the use of knowledge maps for semantic visualization, as such use plays an important role in integrating with the non-relational big data storage of scholarly articles considered for this study. The key features of knowledge maps are listed for a visual representation of knowledge that forms the basis for the proposed model of knowledge discovery of dementia risk factors:

1. Dynamic prediction with new knowledge: A knowledge map representation makes it easy to manage additions to an article repository and identify new knowledge and trends that aid in enhancing prediction and decision-making with reduced time and effort. This feature makes it the best fit for dynamically representing knowledge about modifiable risk factors of dementia updated in the literature.
2. User focuses on new knowledge visually: A knowledge map connects all related data and produces new knowledge based on their relationships established from scholarly articles. This feature brings important but subtle correlations and relationships between dementia risk factors and user focus and attention in a quick manner.
3. Accurate analysis: Knowledge maps can visually reveal unusual or abnormal patterns of scholarly articles for faster detection and human cognition. These patterns highlight hidden data structures relating to dementia risk factors that other types of visual representations might not reveal as well. It is crucial to eliminate such data from a deep analysis to arrive at a more accurate result.
4. Data accessibility in one map: With knowledge maps, more data from several scholarly articles on dementia risks become visually accessible in a single map and are less confusing, thus improving an inference-based performance and productivity for researchers.
5. Faster querying using relationships: Knowledge maps facilitate easier and faster queries because of the relations between the nodes of different dementia risk factors there are visually available.
6. Optimized visual navigation of big data store: A knowledge map can represent a big data store of scholarly articles with optimized visual navigation. When implemented with a non-relational database technique, the results of a user query on dementia risks are not overload with too much data, unlike traditional representations such as tables of rows and columns.
7. Multiple user views: Semantic visualization using knowledge maps facilitates the analysis of data associated with dementia risks from several viewpoints of different types of researchers or data analysts.
8. Filtered topic of interest: A knowledge map can filter information from scholarly articles to show the most appropriate node and its relations along with an interactive navigation. An efficient view of connections between the entities and data organization can identify a specific topic of interest based on the research context of dementia risks matched with the repository.

Overall, the abovementioned features of knowledge maps have been adopted as the basis for proposing a model with a focus on extracting knowledge from scholarly articles. By adopting such a semantic representation of the extracted knowledge embedded as entities and relationships, a mapping to the big data storage of non-relational databases is established. Hence, the proposed modeling of a knowledge map aims to provide an easy navigation for researchers to derive domain-specific evidence-based inferences from scholarly articles.

## Proposed model for semantic visualization

The proposed model is guided by a non-relational database approach that leverages knowledge maps to represent domain-specific knowledge from scholarly articles published in the literature. A semantic visualization not only caters to representing the metadata and contextual content of the articles but also associates semantic descriptions for evidence-based knowledge discovery and inferences. The modeling design follows two key requirements: (1) usability to enable different types of users, including researchers, practitioners, and discipline experts, to derive domain-specific knowledge and (2) scalability to enable growth and contribute to the knowledge base of big data repositories through an automated process that provides flexibility in describing the research contributions.

**Fig. 1:**
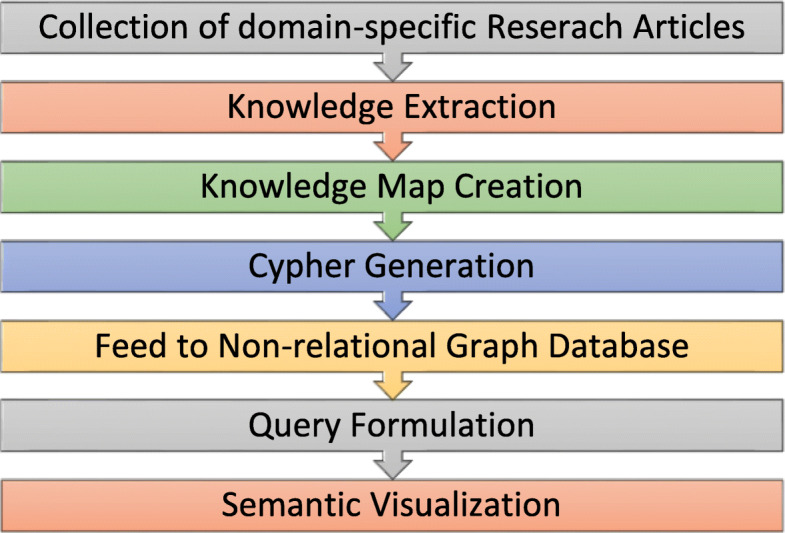
Process flow of the proposed model

The process flow of the proposed model involves seven sequential steps, as shown in Fig. 1. It begins by selecting a set of scholarly research articles that could be sourced from journal repositories in Google Scholar to acquire the required dataset. This is normally achieved using web scrapping tools, resulting in domain-specific articles as required. Next, knowledge is extracted as entities and other knowledge elements from the metadata and relevant content of the research articles. The knowledge extracted from the collection is then utilized to create a knowledge map for a visual presentation using the drawing canvas. Typically, the knowledge elements are various contents of the map, such as ideas, people, documents, data (entities, attributes, relationships), and objectives. These knowledge elements are extracted from scholarly articles using sentence parsing and text mining approaches employing typical metrics, such as the word count and distance measures, to establish the relationship protocols in a visual diagram. Next, a knowledge map diagram with all of the nodes, labels, and property sets is exported to generate the cypher statements to represent all acquired knowledge from the scholarly articles. These advanced cypher statements are pre-processed and inputted into the subsequent step of creating a big data repository using graph theoretic models to build modern visualization methods. The cypher statements are converted into a non-relational graph database schema to establish deep and rich relationships. The pre-processing enforces the merging of new nodes or relationships with existing graph database elements without duplication. In the subsequent step, a query formulation used to discover meaningful knowledge is achieved by translating the required information from the graph database into a cypher query. Finally, these queries of the nodes, properties, and relationships from the graph database are utilized to aid in a semantic visualization.

**Fig. 2:**
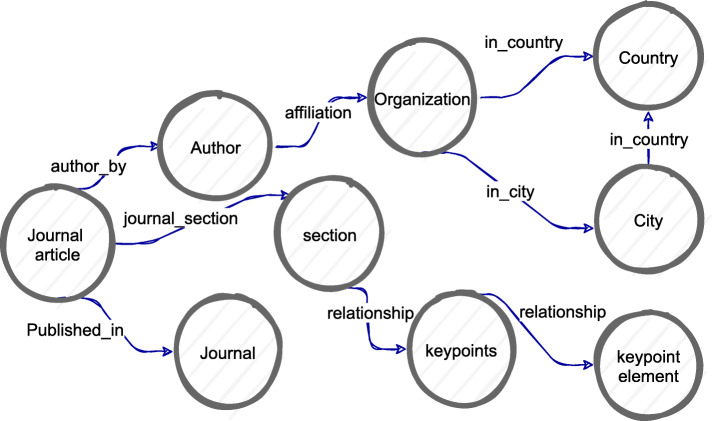
Proposed generic knowledge map modelling for semantic visualization

The proposed generic knowledge map modeling for a semantic visualization of knowledge extracted from scholarly articles is shown in Fig. 2. It shows the visual structure of the knowledge elements and their relationships between journal articles, sections, key points in the sections, authors, affiliations, and publication journal. Entities such as journal articles or titles, authors, journals, and publishers are identified from the metadata. Key knowledge elements are extracted from the content of a collection of research articles and are transformed into entities and relationships that are represented as nodes, circles, and arcs connecting the semantically related knowledge. For the automation of the proposed model for prototype development, Neo4j is adopted, which is a D3.js scripting Arrow tool and Neo4j Bloom application. In Neo4j, circles represent nodes of entities, and arcs represent relationships between the nodes that can be drawn in the Arrow tool as a graph schema diagram. Neo4j stores and queries connected data. The data in Neo4j can be presented in an interactive visual graph environment of Neo4j Bloom.

**Fig. 3:**
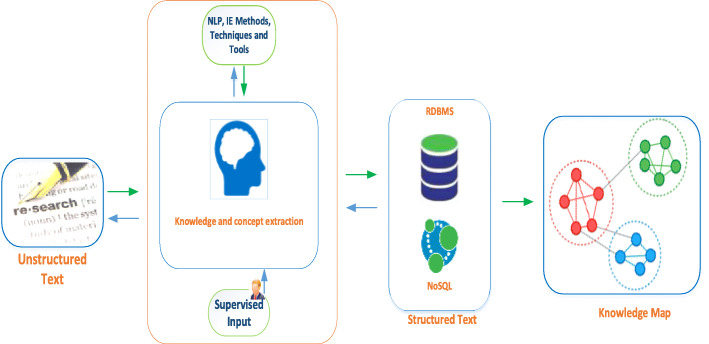
Overall architecture for semantic visualization of unstructured knowledge

The design for automating the entire process flow, illustrated in Fig. 1, requires writing scripts that can create a visual knowledge map with the extracted knowledge elements from scholarly articles. Open-source tools are employed in an integrated manner, and the overall architecture of the proposed modeling approach for a semantic visualization of unstructured knowledge derived from scholarly articles is shown in Fig. 3. Text mining techniques are adopted by using the Arrow tool with the advantage that the knowledge map can be exported in a cypher format using complex JavaScript Object Notation (JSON) documents. They are fed into the Neo4j graph database using advanced cypher scripting statements and are visually presented in Neo4j Bloom. The cypher statements have the advantage of quickly transforming the key data extracted from scholarly articles into a knowledge map structure of rich relationships without duplication of knowledge. Further, the data in Neo4j can undergo semantic querying using various cypher statements to obtain meaningful insights into the big data of scholarly articles when represented as knowledge maps. Such queries can facilitate researchers to synthesize new knowledge by visually integrating and drilling various rich information from the literature. Overall, the proposed semantic querying techniques foster the discovery of new knowledge and their connections, leading to data insight from large collections of articles extremely quickly.

A non-relational graph store is employed as a database for semantic visualization in this study and discussed in detail in a later section, demonstrating the proposed novel approach, which differs from the data visualization in other tools and databases. Neo4j, the data visualization platform adopted here provides a visualization functionality to display a knowledge map representation of the data saved in a relational database management system with the help of the cypher query.

Knowledge map constructs can be weak or strong depending on the use of spatial relationships and beyond. Entity properties and their spatial relations are to be determined using a good quantitative/qualitative metric, which can include the distance measures, costs, and weights. A recent study developed a computationally lightweight score based on regression analysis to measure the importance of journals without requiring any data storage [37]. Further, delay differential equations were used to analyze the cause and control variables responsible for the growing influence of a journal using visual knowledge maps [38]. However, the aim of the proposed semantic visualization using knowledge maps is more for knowledge discovery and evidence-based inference, rather than merely calculating a journal influence score or estimating the influence of the journal. The aim here is to establish relationships that include spatial, chronological, hierarchical, associative, causal, logical, and evaluative properties for a visual representation of strongly descriptive knowledge maps. A graph theory approach is adopted with spatial aspects such as adjacency, distance, and containment to create knowledge maps and to store and retrieve the data with an optimized search. The details of mathematical modeling along with the application of graph theory in Neo4j adopted in this research are provided below.

### Knowledge extraction

The data modeling within the knowledge map construct includes the main knowledge elements extracted from the research articles, which are represented as nodes and other elements showing the relationship between the knowledge elements and are represented as links or arrows. The knowledge elements comprise metadata of the research article and other relevant content including key highlights. Each knowledge element is a unique word or phrase in the context of the research article. Therefore, mathematically, a knowledge map (M) is denoted that can be defined as a set of knowledge elements (K) and relationships (R) among knowledge elements, that is,

M = (K, R, ϕ), along with the following:

- K ⊆ M and R ⊆ M.
- K is a set whose elements are called knowledge elements and represents nodes in the knowledge map, that is, K = {k_1_,k_2_,k_3_,k_3_,…,k_*n*_}, where.
  - k_y_ ∈ K and 1 ≤ *y* ≤ *n*, where *n* is the total number of words or phrases extracted from the research article.
- R is a set whose elements are called relationships among the knowledge elements and represents arcs or links in the knowledge map, that is, R = {r_1_,r_2_,r_3_,……,r_*n*_}.
- ϕ: R → {(*x*, *y*) | (*x*, *y*) ∈ K^2^ ∪ *x* ≠ *y*}, where the following hold:
  - ϕ represents a set of relationships (R), which are ordered pairs of coupled knowledge elements (K).
  - In the relationship (*x*, *y*) directed from *x* to *y*, the knowledge elements *x* and *y* are called the endpoints of the relationship, *x* is the start node of the relationship, and *y* is the end node of the relationship.
  - A knowledge element only exists in a knowledge map if it belongs to a relationship.

These notations are formalized for a particular research article ‘a’ in developing the set of all relationships ϕ_a_ among the knowledge elements within a knowledge map. In addition, R_a_ = {r_a1_, r_a2_, r_a3_, …, r_am_} is used such that ordered pairs of knowledge elements with the mathematical mapping resulting in the entire set of relationships for article ‘a’ is given by ϕ_a_: r_a1_ → {(k_a1_, k_a2_) | (k_a1_, k_a2_) ∈ K^2^ ∧ k_a1_ ≠ k_a2_}.

The Arrow tool is adopted to create a visual graph database schema model and automate the implementation using Neo4j cypher scripts to generate the database schema with database entities and relationships. The knowledge elements and relationships are stored and queried as a non-relational database schema representing big data repositories, as described in the following.

### Non-relational database in big data using Neo4j

Neo4j, a non-relational database for the proposed prototype implementation from a big data repository is employed because it provides native graph data storage, processing tools, and visualization tools required for semantic visualization using knowledge maps. The graph theoretic approach of the proposed model is incorporated using Neo4j to store and retrieve the knowledge as non-relational data and to optimize the search by using the index-free adjacency technique. In Neo4j, the data and relationship between the data are represented by graph structures consisting of nodes, labels, properties, and links [39]. The following three key advantages of the graph-based not only structured query language (NoSQL) database over traditional relational databases are capitalized for this research:

- The NoSQL database schema can be defined or altered dynamically at run-time.
- The relationships between the nodes are objects in the database, which can be queried directly.
- The properties can be associated with nodes as well as relationships and can be queried directly.

According to ref. [40], Neo4j is the most popular non-relational graph database, and is optimized for mapping data points and connecting links between nodes and relationships visually. The main domain-specific knowledge and entities from the research articles are represented as nodes, and the semantics that relate to the knowledge elements are represented along the arcs. Neo4j has been widely adopted in various niche applications such as artificial intelligence, machine learning, Internet of Things, real-time recommendation systems, fraud detection, network and IT operations, and identity and access management [3]. For the proposed model, because nodes representing knowledge may have multiple semantic associations with other nodes, which in turn can have multiple properties and labels, Neo4j supporting these characteristics forms the best fit. The properties are data attributes of each knowledge element, such as author names, gender, and affiliation, extracted from the scholarly articles. The labels are the names of the group nodes that categorize the domain of the nodes, and these are used to find nodes in the graph in queries. The cypher query language (CQL) of Neo4j is adopted to create, query, or traverse the graph with properties of the nodes and relationships [41, 42]. Scripting constructs such as ‘Match’, ‘Where’, and ‘Return’, as well as command operator signs such as ( ), [ ], { }, -, and -> in CQL are closer to the search clauses and commands predominantly employed by users as compared to structured query language. Therefore, CQL can be effectively used to automate the retrieval and querying of knowledge stored in a graph database. In the next section, the implementation of the proposed model in Neo4j is described for dementia risk factors as a case study.

## Model implementation for case study and results

The collection of research articles about the risk of dementia is considered for the application of the proposed model as a case study. Some existing studies have focused on knowledge graph creation from scientific literature on degenerative diseases to help researchers investigate their discovery relationships with the discoveries or domain knowledge of other researchers [43]. However, such implementations reported in the literature suffer from challenges such as the automatic generation of a semantic visualization and mapping of secondary information related to the main knowledge elements. The proposed model implementation overcomes these limitations in existing studies. Further, focus is on the domain of research investigations concerning the risk factors of dementia for supporting researchers and practitioners in deriving knowledge on recently reported scholarly articles on modifiable risk factors owing to varying environmental and other factors that can affect the timely diagnosis and follow-up decisions in healthcare [3, 9]. It will therefore be useful to create an automatic repository of domain-specific knowledge from scholarly articles related to the risk factors of dementia, allowing researchers to quickly discover knowledge and promote further research on this important topic.

For application of the proposed model to scholarly articles on dementia risk factors, the following open-source software tools are adopted through the integration of the following scripting programs:

1. Arrow tool to create a visual graph and generate the Neo4j cypher statements for nodes and relationships to establish complex hierarchies of knowledge related to dementia risk factors. From the complex JSON documents extracted from scholarly articles, the cypher statements deconstruct and transform them into a knowledge map structure of rich relationships.
2. Neo4j Community server with Neo4j browser to automatically create a graph database using the generated cypher statements from Arrow for indexing and querying the non-relational database with the terms of dementia risk factors.
3. The Neo4j Bloom application is available using the database server to explore the graph and visually interact and present the graph results.

**Fig. 4:**
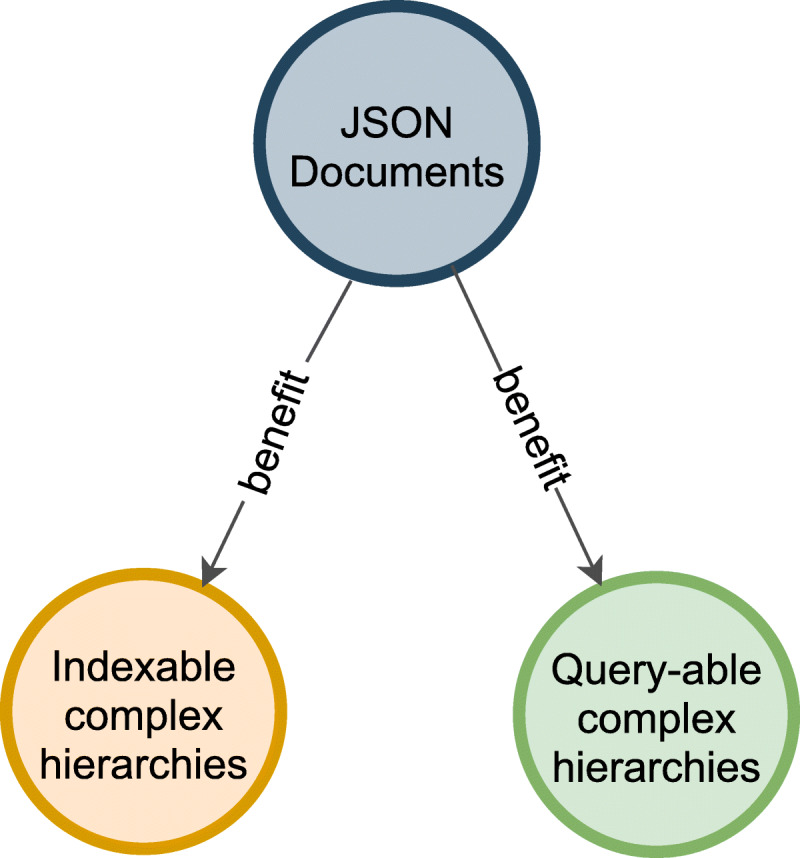
High-level architecture to automate the complex hierarchies

A high-level architecture for automating the complex hierarchies with JSON documents, NoSQL indexing, and querying is shown in Fig. 4, illustrating the beneficial relationship of the articles. In addition, as the advantage of the proposed software platform, it is standards-based and interoperable because there are multiple visualization tools that handle the exported data from Neo4j by generating cypher queries to create data in Neo4j databases.

A demonstration is provided using a sample of unstructured text collected from research articles to build a prototype dataset to show the significance and effectiveness of a semantic visualization in the form of graph databases and knowledge maps. The following is the first sample text taken from one of the research articles from the collection of research articles related to risk factors for dementia [3]:

Our study found a potentially important effect modification between exercise and physical functioning in …[which does] not prevent dementia but might be associated with a delay in onset. If these post hoc findings are confirmed, senior citizens may have more reason to...

**Fig. 5:**
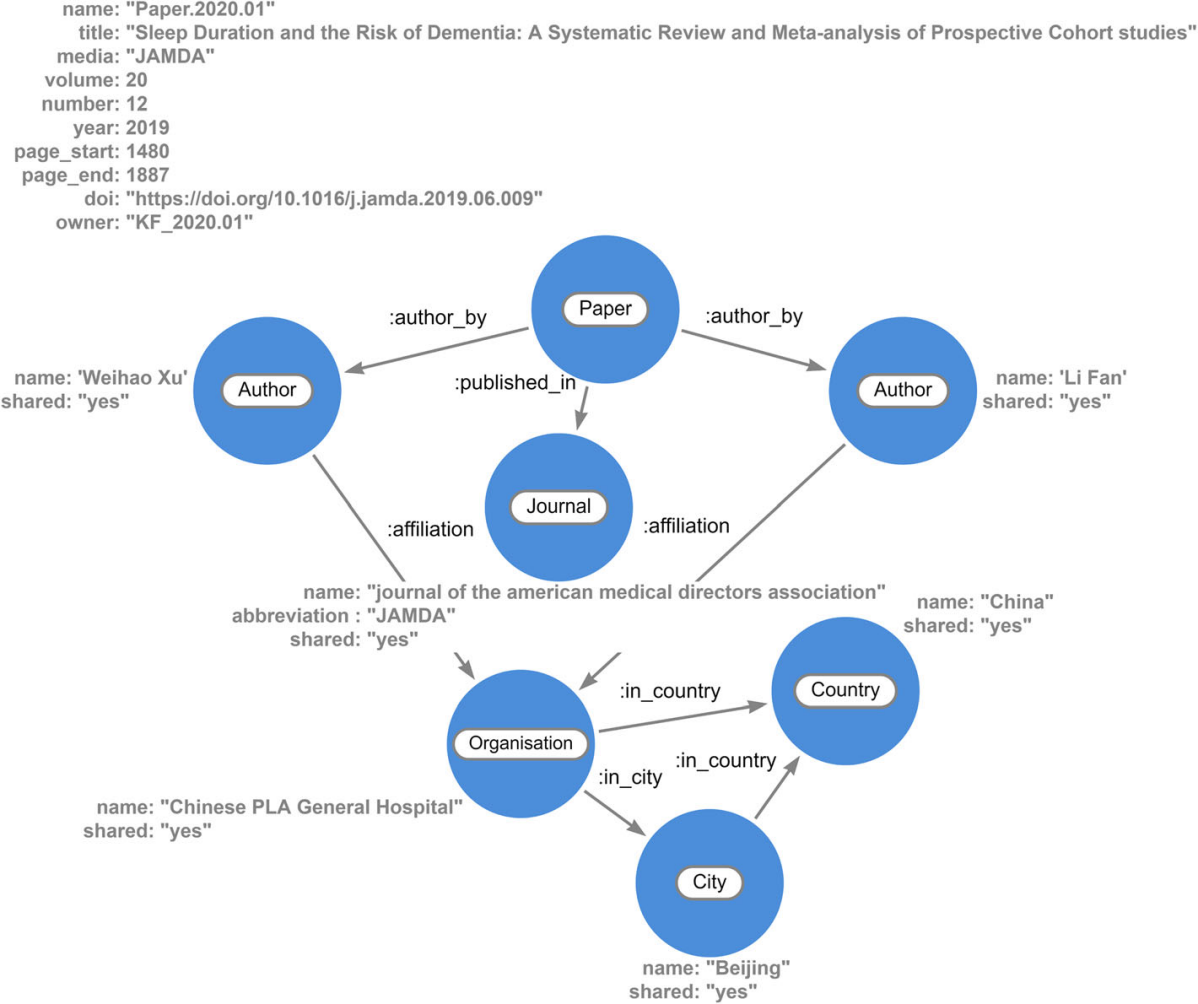
Snapshot of knowledge extracted to outline conceptual data model using Arrow tool

The highlights and key points that are available as unstructured text in the research article are considered for text mining, as shown in Fig. 2. Next, the Arrow tool map is employed, as shown in Fig. 5, to rapidly sketch the graph database schema based on the proposed data model for achieving a semantic visualization. The Arrow tool is an intuitive way to draw the nodes and relationships with properties to capture the key highlights of the research articles. The nodes, directed relationships, and their properties are identified and made by utilizing the drag-drop interface of the tool for each key point of the research articles. It is simply a visual representation of a graph with nodes and arcs and without any permanent data storage of the underlying knowledge. However, a visual representation of a knowledge map is useful for manually verifying the knowledge extracted to establish the correctness of the proof-of-concept implementation of the proposed model for the collection of sample research articles related to dementia. Figure 5 demonstrates a semantic visualization of the sample knowledge extracted using the text mining technique and the above-mentioned graph theory-based data modeling.

**Fig. 6:**
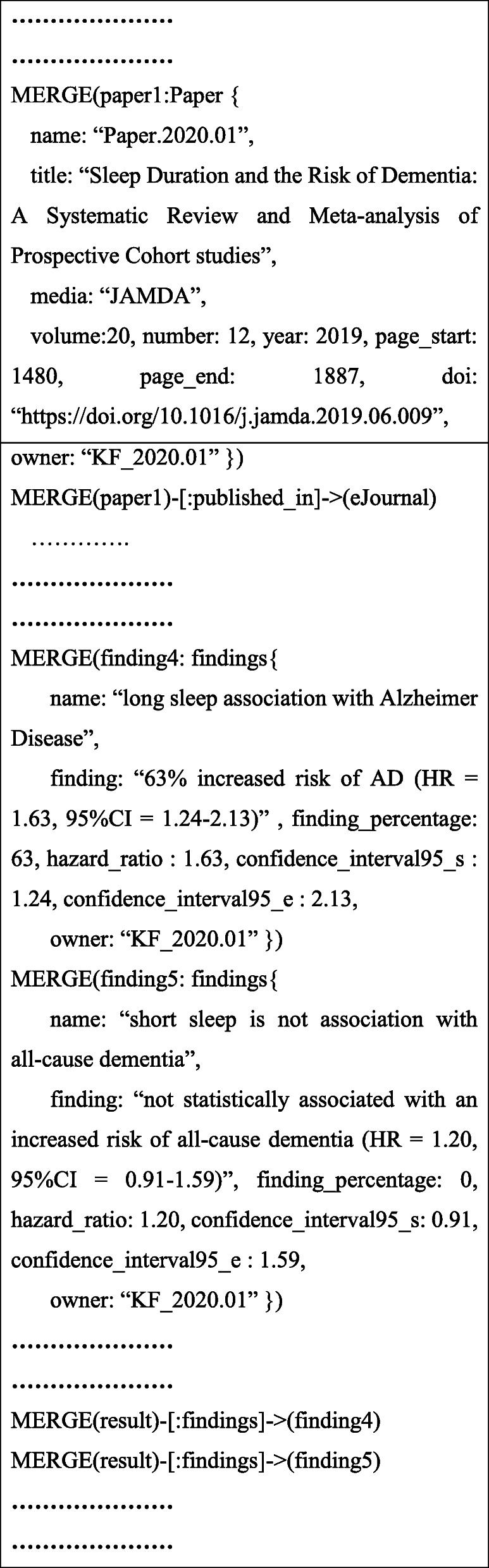
Snapshot of cypher statements for dementia risk factor case study

The knowledge map drawn using the Arrow tool shown in Fig. 5 is based on the knowledge extracted from the articles and can be exported into various formats to achieve a permanent storage of the big data collected. In this proof-of-concept implementation, the knowledge map is exported as cypher statements to include all labels and properties of the nodes and relationships, which are executed in Neo4j to form a graph datastore. Figure 6 shows a snapshot of the cypher statements that connect key information from the text of a scholarly article related to dementia risk factors. A similar process is adopted in exporting the conceptual knowledge design of Arrow tool from the research articles into cypher statements.

**Fig. 7:**
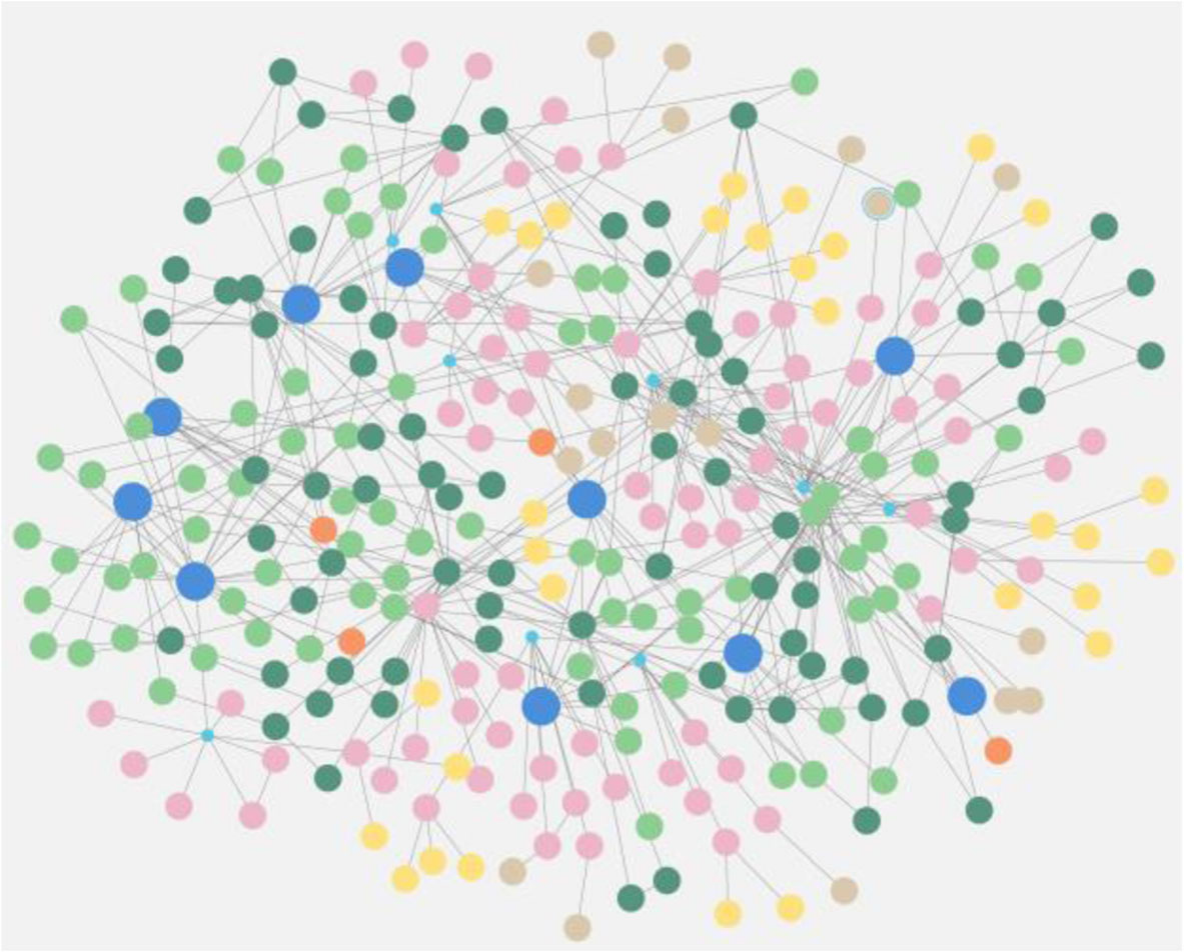
Visual knowledge map in Neo4j graph database using Neo4j Bloom

Figure 7 shows the final visual output achieved after the conceptual design of the Arrow tool is exported as a cypher statement, which are executed in Neo4j to create a graph database. It provides a high-level visual representation of a graph database with the nodes and the complex relationships of the knowledge extracted from the research articles considered in this study. In the case of dementia risk factors, Fig. 7 presents an overview of a graph database generated with 295 nodes and 473 relationships among the nodes to show that interconnected data can be traversed to support user query and data processing to achieve a semantic visualization. The relationship can be displayed by selecting tags in the node labels and relationship types, and each node can expand the links to other connections that are available, thereby facilitating a visual navigation of the big data storage for knowledge discovery.

The CQL match statement in Neo4j is used to retrieve all nodes and their relationships as a high-level visual knowledge map of the graph database portrayed in Fig. 7. A typical CQL match statement is as follows:

$$MATCH\left(n\right)-\left[r\right]-\left(m\right)\;RETURN\;n,r,m$$

**Fig. 8:**
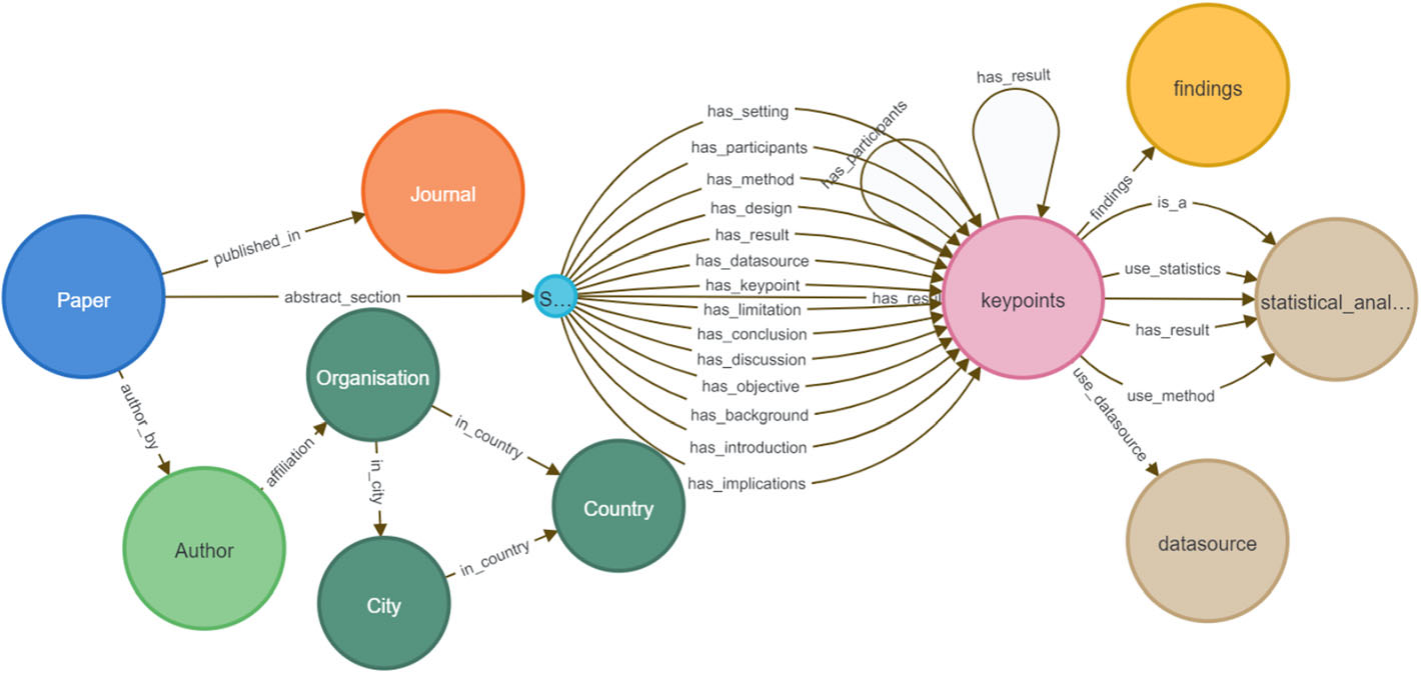
Visual data model of knowledge map for the case of dementia risk

The data model implemented for the case study of dementia risk factors from scholarly articles is shown in Fig. 8. The following cypher statement is executed to retrieve the underlying schema of the database in Neo4j:

$$call\;db.schema.visualization$$

The proposed generic conceptual data model shown in Fig. 2 is applied to develop a semantic visualization for the dementia risk factor case study. The proof-of-concept of the graph-based big data store is implemented to store the knowledge of dementia risk factors extracted from a small collection of scholarly articles. This is based on the cypher statements representing the schema of the designed database, as shown in Fig. 6. Color-coding of the nodes was set in the Neo4j data model to match and identify the entities visually, as shown in Figs. 7 and 8. For example, a blue node indicates an article as a paper entity in the graph database, and orange indicates the journal where the article was published. The color scheme is pre-determined by the Neo4j software to uniquely distinguish the knowledge elements. The use of colors helps to clearly identify and differentiate knowledge within a visual knowledge map.

**Fig. 9:**
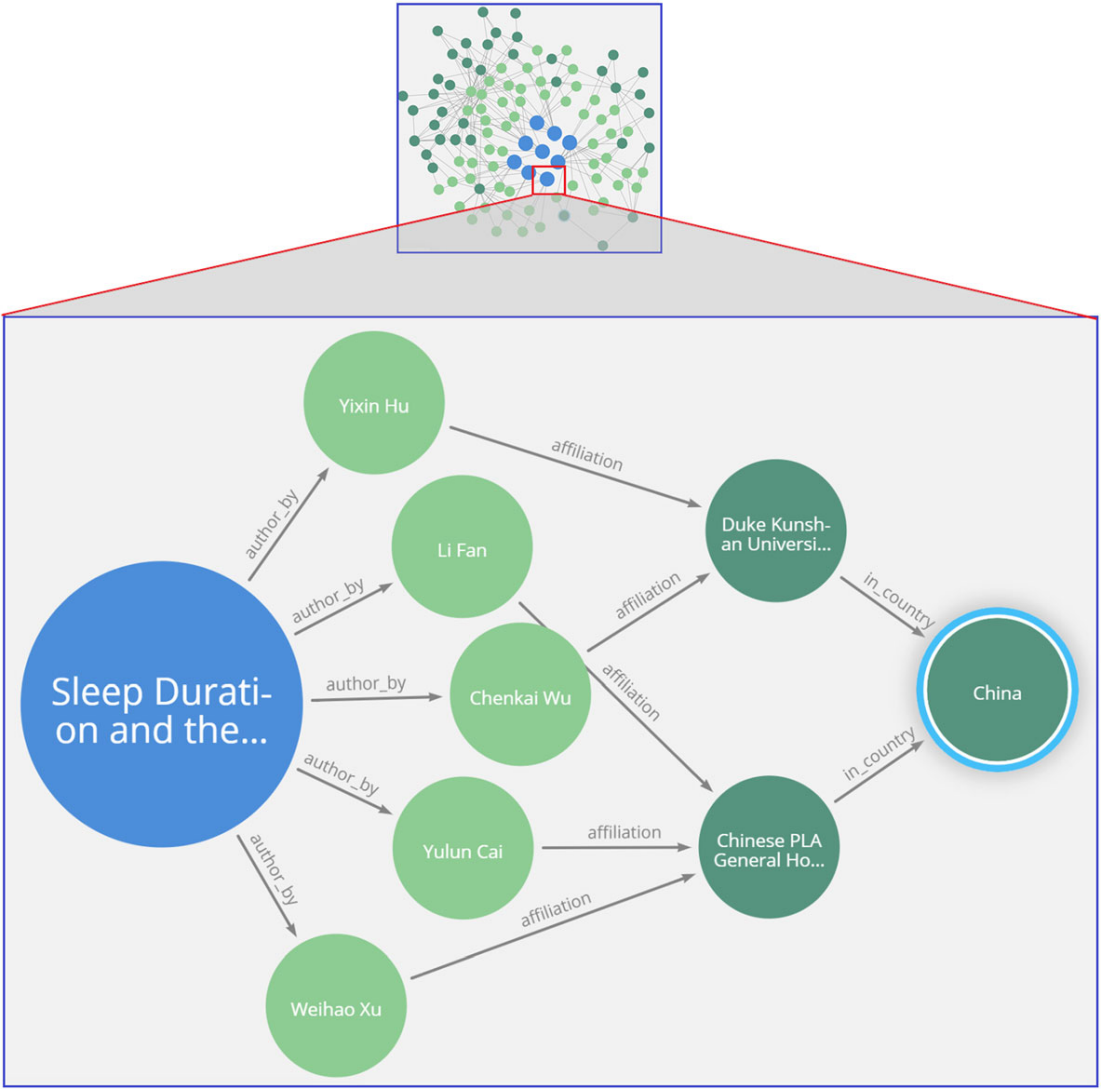
Knowledge map of database query finding authors of research articles with their affiliations and country

**Fig. 10:**
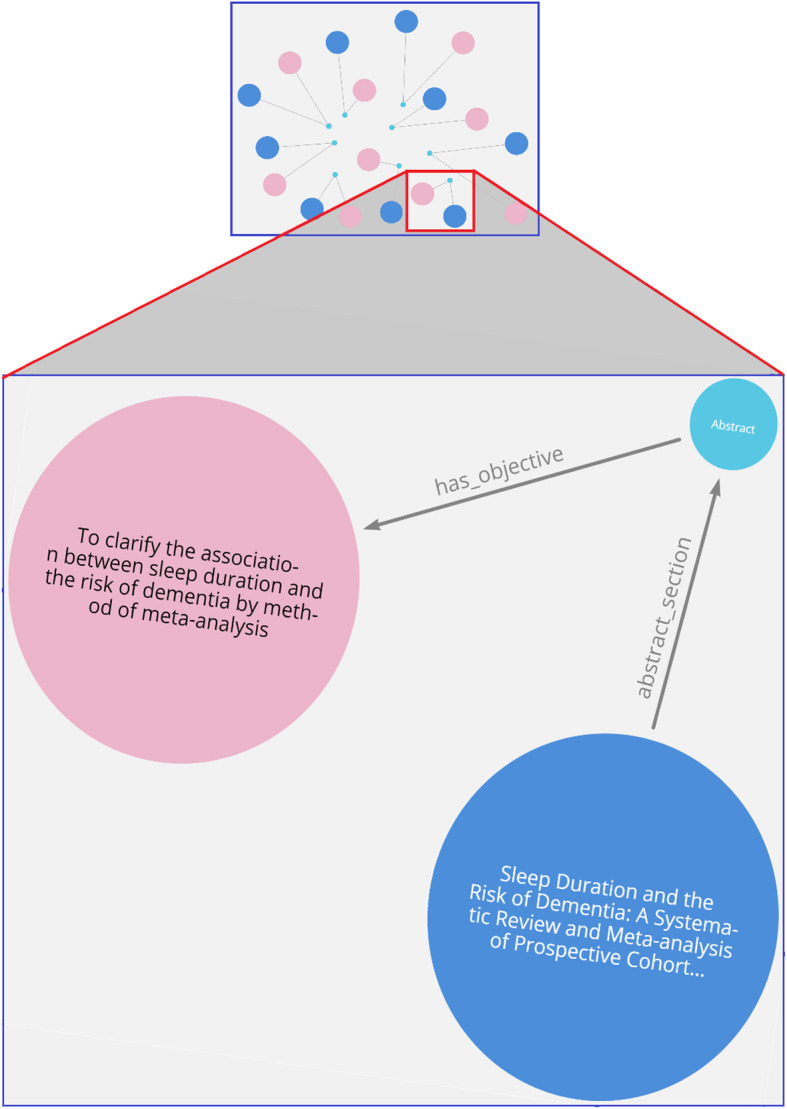
Knowledge map visualization used to identify objectives of journal articles

**Fig. 11:**
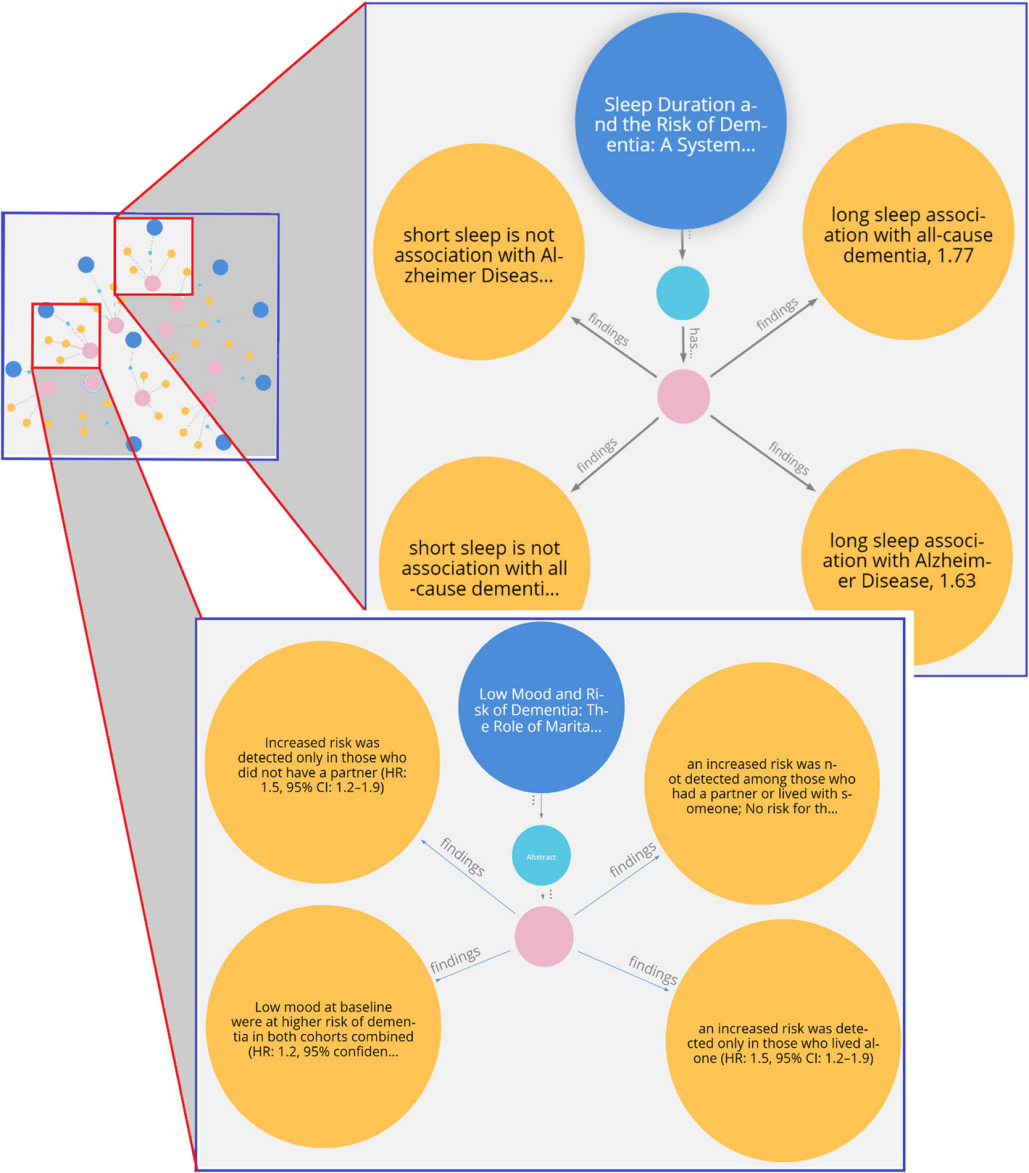
Knowledge map of dementia risk factors for knowledge discovery

The graph database developed for storing knowledge related to dementia risk factors extracted from articles can be visually traversed to explore and search the data, as well as query the interrelationships among the data to gain deeper insight. The output data when visualized as a color-coded knowledge map helps in understanding the interconnected structure of the database and facilitates an exploration of different perspectives for knowledge discovery. Numerous visualizations are possible using the proof-of-concept graph database implemented with the proposed conceptualization model. The following key semantic visualization scenarios are provided:

1. A knowledge map of authors working within the dementia domain that can be further filtered with queries to find those authors working on a particular risk factor (e.g., Fig. 9). This knowledge can benefit researchers to find common author backgrounds, such as the affiliation and country, for possible research collaboration.
2. A knowledge map of papers with common objectives focused on dementia risk factors (e.g., Fig. 10). This is useful for researchers to interrelate any two papers based on their common research objectives.
3. A knowledge map of a specific risk factor of dementia with drill-down queries providing deep data insights and knowledge discovery of research findings (e.g., Fig. 11).

Figure 9 displays a network of journal articles as a knowledge map and shows a zoomed-in image of the authors who have researched dementia risk factors, such as sleep duration. The semantic visualization helps query the authors’ affiliations and countries from the graph database. The blue circles represent article journal nodes, and the light green nodes are the authors connected by arcs. This knowledge map of connected data shows the contribution trends of institutions and countries in the domain of dementia, which can help researchers to establish collaborations. This visual mapping is displayed in Neo4j Bloom without the need to create any CQL statement. However, in the absence of a knowledge map, a traditional approach is to run CQL statements on Neo4j to retrieve the required information. To make a query on the database for finding authors, as well as their affiliation and country, based on an article title related to ‘dementia’, a researcher should know how to create and run the following command:

$$\begin{array}{*{20}c}MATCH(p:Paper)-\lbrack r1:author\_by\rbrack-(a:Author)-\lbrack r2:affiliation\rbrack-(o:Organization)-\lbrack r3:in\_country\rbrack-(c:Country)WHEREp.title=\sim'\ast Dementia\ast'\\RETURN\;p,r1,a,r2,o,r3,c\end{array}$$

Hence, researchers would likely prefer to adopt knowledge maps for ease of use.

Figure 10 shows a knowledge map of journal articles with research objectives relating to common dementia risk factors, such as sleep duration. The blue-colored nodes represent the journal, and the pink-colored nodes represent the objectives of the articles. This mapping can help in identifying the research objective trends in the dementia domain, particularly on its risk factors. The cypher query designed to create the search in the Neo4j Bloom is given below:

$$MATCH(p:Paper)-\lbrack r1:abstract\_section\rbrack-(s:Section)-\lbrack r2:has_\_objective\rbrack-(k:keypoints)where(k.objective)Return\;p,r1,s,r2,k.objective\;UNION\;MATCH(p:Paper)-\lbrack r1:abstract\_section\rbrack-(s:Section)-\lbrack r2:has\_introduction\rbrack-(k:keypoints)where(k.objective)Returnp,r1,s,r2,k.objective$$

In Fig. 11, the knowledge map shows how a researcher can gain deep insight into specific risk factors for dementia using a visual-friendly navigation. Visual mapping displays the risk factors for dementia and research findings with the hazard ratio reported in journal articles and their relationships. The cypher statements designed to create the required filtering in the Neo4j Bloom are given below:

$$MATCH\;(p:Paper)-\lbrack r1\rbrack-(s)-\lbrack r2\rbrack-(k:keypoints)-\lbrack r3:findings\rbrack-(f:findings)\;RETURN\;p,r1,s,r2,k,r3,f$$

The relationship connecting the nodes in a knowledge map specifies the association between nodes, and it clearly identifies semantics, such as which knowledge pertaining to a dementia risk factor belongs to which paper publication or which author. As the most beneficial aspect of using a graph database, any addition or modification of links between objects can take place dynamically using a visual navigation. Further, there are no limitations for adding relationships, and the knowledge map can display relationships between entities and other nodes based on what the user wishes to query. For example, queries such as who/what influenced whom/what, what are the benefits of *X*, and the terms of the relationships can be determined interactively through a few clicks on the knowledge elements displayed. Figure 10 illustrates the convenience provided to a researcher in interpreting the outcome of complex queries through a semantic visualization using automatic CQL scripts that are generated from knowledge map traversals. Without such knowledge maps, it would be time consuming for researchers and practitioners to read the text from various articles and interpret them. For example, identifying the risks of dementia associated with short sleep duration reported in research articles would require an extensive literature review. Neo4j includes color and font settings for a visual layout that enhances the ease of data categorization allowing users to easily interpret the knowledge.

Overall, Figs. 7 and 8 demonstrate the successful application of the proposed model for a semantic visualization used to implement a graph-based datastore as a prototype. The convenience of a visual knowledge map navigation is also demonstrated using Figs. 9, 10 and 11, which display the outcomes of user queries facilitating knowledge discovery from the graph datastore. Examples of CQL statements provide snapshots of automating the processes. Using interactive queries through a graph navigation, knowledge discovery about dementia risk factors was successfully illustrated from a sample scholarly article considered as a proof-of-concept implementation. The case study for this purpose considered scenarios such as determining a long sleep duration as a risk factor of dementia or identifying low mood as an increased risk of dementia.

**Fig. 12:**
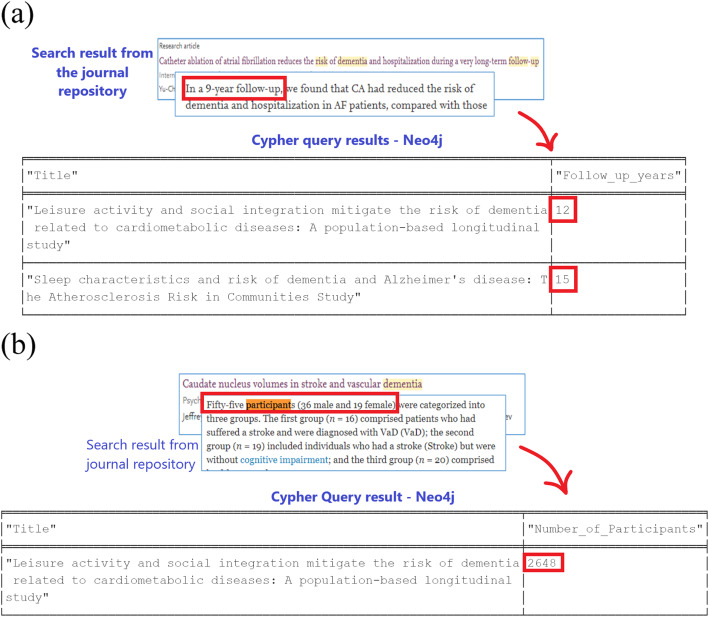
(**a**): Comparison of search results to identify research articles related to the risk of dementia where the number of follow up years is greater than 10; (**b**): Comparison of search results to identify research articles related to risk of dementia where the number of research participants is within 2000–2900

In addition to the visualization of the knowledge map, the outcomes of a cypher query automation from the repository provide more accurate results than search functions in journal repositories such as ScienceDirect. For example, to find a collection of research articles studying the risk of dementia with a follow-up of greater than 10 years, the phrase “risk of dementia follow up years greater than 10” was used for a simple comparative benchmarking. The search results from the ScienceDirect repository are compared with the resulting set of a cypher queries executed in the proof-of-concept implementation of the proposed modeling as a big data graph repository. The ScienceDirect search results contained a few incorrect results, whereas the graph database provided accurate results for cases in which the number of follow-up years was greater than 10, as shown in Fig. 12(a). A similar comparison was adopted by using the search phrase “risk of dementia number of participants between 2000 and 2900” to find research articles on the risk of dementia in which the number of participants was greater than 2000 and less than 2900. Figure 12(b) shows the inaccurate research article(s) returned by a search in the ScienceDirect repository. However, the proposed proof-of-concept returned accurate results using the model implementation of knowledge maps, which facilitated a visual navigation of the graph datastore.

Overall, how a knowledge map can be interrogated interactively was demonstrated. The proposed generic semantic visualization model using graph data resources will aid researchers in the dynamic exploration of knowledge discovery. More information regarding the node on “sleep duration” and its link to a central ‘exercise’ node or other dementia risk factors can be queried. Further drilling to identify what types of exercise are important (e.g., cardiovascular vs strength-based) can be conducted if reported in the articles. One can view or hide the information given to neighboring nodes with the ease of a single click. A recent study reported limited applications of knowledge maps found in the literature because very few studies have addressed the issues of their implementation and user evaluation [44]. The present study is a modest step to fill in this gap within the literature. In line with other studies, user acceptance of the proposed proof-of-concept implementation is theoretically based on the features of perceived usefulness and perceived ease of use. The structural equation modeling adopted with the proposed cypher statements was validated using the underlying technology acceptance model. Hence, theoretical and empirical investigation approaches were adopted to verify the correctness of the system developed in this study.

## Conclusions and future research

In this paper, the initial steps are presented for enhancing knowledge discovery from scholarly articles with an effective use of the knowledge mapping technique for a semantic visualization combined with the non-relational big data paradigm. Various types of semantic visualization techniques were discussed, and the significance of the knowledge maps was established. The proposed model for automatic knowledge extraction, representation, and query formulation was developed to achieve a semantic visualization of domain-specific information to facilitate evidence-based knowledge discovery and inference. The architecture used to automate the process flow, and the implementation of the model as a prototype for knowledge discovery from scholarly articles on dementia risk factors, were presented.

The results of the proposed semantic visualization model implementation were illustrated using knowledge maps representing a non-relational database with case scenarios. The proof-of-concept prototype of the case study of dementia risk factors captured unstructured text from the literature in a big data environment and demonstrated the significance of a semantic visualization. The effective use of knowledge maps interactively showcased the ease in knowledge discovery, which would be beneficial for researchers working in the knowledge domain, saving much time and effort.

Overall, the effectiveness of the prototype was demonstrated through meaningful knowledge extraction and analysis from unstructured data using the Neo4j graph database in the context of dementia risk factors as a case study. The use of an emerging NoSQL database technology such as Neo4j was justified, the features of which were exploited to visually represent both the graph datastore as a knowledge repository as well as user queries of the database by applying graph theoretic modeling. Visual navigation was achieved by generating strong and effective knowledge maps. This work has advanced research in the direction of semantic visualization using knowledge maps, taking the first steps to pave the way for a larger research agenda.

The limitation of this research study is the small number of research articles utilized to implement and demonstrate the prototype. However, the proposed model and implementation approach can be easily applied to any larger collection, which is planned for future studies. In addition, theoretical and empirical approaches were employed to validate the proof-of-concept implementation. Future research will involve developing a comprehensive knowledge repository for exploring many other domain-specific visual knowledge discoveries with more advanced deep mining tools and quality evaluation metrics. 

## Data Availability

The database and scripting programs developed in this paper is not open, but authors can provide on demand.

## Abbreviations

CQL: Cypher query language
ICT: Information and communication technology
JSON: JavaScript Object Notation
NoSQL: Not only structured query language

## References

[1] De Mauro A, Greco M, Grimaldi M (2016). A formal definition of Big Data based on its essential features. Libr Rev.

[2] Paulheim H (2017). Knowledge graph refinement: a survey of approaches and evaluation methods. Semant Web.

[3] Peters R, Booth A, Rockwood K, Peters J, D’Este C, Anstey KJ (2019). Combining modifiable risk factors and risk of dementia: a systematic review and meta-analysis. BMJ Open.

[4] Aslam MA, Aljohani NR, Abbasi RA, Lytras MD, Kabir MA (2017) A generic framework for adding semantics to digital libraries. In: Ciuciu I, Debruyne C, Panetto H, Weichhart G, Bollen P, Fensel A, et al (eds) On the move to meaningful internet systems: OTM 2016 workshops. Confederated international workshops: EI2N, FBM, ICSP, Meta4eS, and OTMA 2016, October 2016. Lecture notes in computer science, vol 10034. Springer, Cham, pp 277–281. 10.1007/978-3-319-55961-2_28

[5] Aryani A, Wang JB (2017) Research graph: building a distributed graph of scholarly works using research data switchboard. In: Abstracts of open repositories conference, Brisbane, 27 June 2017

[6] Burton A, Koers H, Manghi P, Stocker M, Fenner M, Aryani A et al (2017) The scholix framework for interoperability in data-literature information exchange. D-Lib Mag 23(1–2). 10.1045/january2017-burton

[7] Bechhofer S, De Roure D, Gamble M, Goble C, Buchan I (2010) Research objects: Towards exchange and reuse of digital knowledge. Nat Preced, arXiv:1901.10816. 10.1038/npre.2010.4626.1

[8] Jaradeh MY, Oelen A, Farfar KE, Prinz M, D’Souza J, Kismihók G et al (2019) Open research knowledge graph: next generation infrastructure for semantic scholarly knowledge. In: Abstracts of the 10th international conference on knowledge capture, ACM, Marina Del Rey, 19–21 November 2019. 10.1145/3360901.3364435

[9] Larson EB, Wang L, Bowen JD, McCormick WC, Teri L, Crane P (2006). Exercise is associated with reduced risk for incident dementia among persons 65 years of age and older. Ann Intern Med.

[10] Armstrong JJ, Mitnitski A, Andrew MK, Launer LJ, White LR, Rockwood K (2015). Cumulative impact of health deficits, social vulnerabilities, and protective factors on cognitive dynamics in late life: a multistate modeling approach. Alzheimers Res Ther.

[11] Godin J, Armstrong JJ, Rockwood K, Andrew MK (2017). Dynamics of frailty and cognition after age 50: why it matters that cognitive decline is mostly seen in old age. J Alzheimers Dis.

[12] Hanson KL, DiLauro T, Donoghue M (2015) The RMap project: capturing and preserving associations amongst multi-part distributed publications. In: Abstracts of the 15th ACM/IEEE-CS joint conference on digital libraries, ACM, Knoxville, 21–25 June 2015. 10.1145/2756406.2756952

[13] Sadeghi A, Lange C, Vidal ME, Auer S (2017) Integration of scholarly communication metadata using knowledge graphs. In: Kamps J, Tsakonas G, Manolopoulos Y, Iliadis L, Karydis I (eds) Research and advanced technology for digital libraries. 21st international conference on theory and practice of digital libraries, TPDL 2017, September 2017. Lecture notes in computer science, vol 10450. Springer, Cham, pp 328–341. 10.1007/978-3-319-67008-9_26

[14] Shneiderman B (1994). Dynamic queries for visual information seeking. IEEE Softw.

[15] Kim J, Vasardani M, Winter S (2016). From descriptions to depictions: A dynamic sketch map drawing strategy. Spat Cogn Comput.

[16] Vacek M, Krbalek P (2012) Semantics of knowledge map visualization. Paper presented at the 12th WSEAS international conference on applied informatics and communications, Istanbul, 21–23 August 2012

[17] Chen CR, Lin Y (2012) Enhancing knowledge management for engineers using mind mapping in construction. In: Hou HT (ed) New research on knowledge management technology. IntechOpen, Rijeka. 10.5772/34515

[18] Akhavan P, Pezeshkan A (2014). Knowledge management critical failure factors: A multi-case study. VINE.

[19] Balaid ASS, Zibarzani M, Rozan MZA (2012). A comprehensive review of knowledge mapping techniques. J Inform Syst Res Innov.

[20] Lv YJ, Zhao G, Miao P, Guan YJ (2013). Construction of intelligence knowledge map for complex product development. J Eng Sci Technol Rev.

[21] Davies M (2011). Concept mapping, mind mapping and argument mapping: what are the differences and do they matter?. High Educ.

[22] Gruber TR (1993). Toward principles for the design of ontologies used for knowledge sharing?. Int J Hum Comput Stud.

[23] Rousseau D, Billingham J, Calvo-Amodio J (2018). Systemic semantics: a systems approach to building ontologies and concept maps. Systems.

[24] Stringfield SG, Luscre D, Gast DL (2011). Effects of a story map on accelerated reader postreading test scores in students with high-functioning autism. Focus Autism Other Dev Dis.

[25] Leung NKY, Kang SH, Lau SK, Fan J (2009). Ontology-based collaborative inter-organizational knowledge management network. Int J Inform Knowl Manage.

[26] Abbasi AA, Kulathuramaiyer N (2016). A systematic mapping study of database resources to ontology via reverse engineering. Asian J Inform Technol.

[27] Meštrović A, Calì A (2016) An ontology-based approach to information retrieval. In: Calì A, Gorgan D, Ugarte M (eds) Semantic keyword-based search on structured data sources. COST action IC1302 second international KEYSTONE conference, IKC 2016, September 2016. Lecture notes in computer science, vol 10151. Springer, Cham, pp 150–156. 10.1007/978-3-319-53640-8_13

[28] Hazber MAG, Li RX, Gu XW, Xu GD (2016). Integration mapping rules: transforming relational database to semantic web ontology. Appl Math Inform Sci.

[29] Saha D, Floratou A, Sankaranarayanan K, Minhas UF, Mittal AR, Özcan F (2016). ATHENA: an ontology-driven system for natural language querying over relational data stores. Proc VLDB Endowm.

[30] Calvanese D, Cogrel B, Komla-Ebri S, Kontchakov R, Lanti D, Rezk M (2017). Ontop: answering sparql queries over relational databases. Semant Web.

[31] Munir K, Anjum MS (2018). The use of ontologies for effective knowledge modelling and information retrieval. Appl Comput Inform.

[32] Maynard D, Lepori B, Petrak J, Song XY, Laredo P (2020). Using ontologies to map between research data and policymakers’ presumptions: the experience of the KNOWMAK project. Scientometrics.

[33] Balaid A, Rozan MZA, Hikmi SN, Memon J (2016). Knowledge maps: A systematic literature review and directions for future research. Int J Inform Manage.

[34] Rao LL, Mansingh G, Osei-Bryson KM (2012). Building ontology based knowledge maps to assist business process re-engineering. Decis Support Syst.

[35] Lee JH, Segev A. Knowledge maps for e-learning. Comput Educ 59(2):353–364. 10.1016/j.compedu.2012.01.017

[36] Ginde G (2016) Visualisation of massive data from scholarly article and journal database: a novel scheme. Presented at PESIT Bangalore South Campus, Bangalore, arXiv:1611.01152

[37] Saha S, Jangid N, Mathur A, Narsimhamurthy AM (2016). DSRS: estimation and forecasting of journal influence in the science and technology domain via a lightweight quantitative approach. COLLNET J Scientometr Inform Manage.

[38] Saha S, Sarkar P, Mathur A, Basak S (2018). Model visualization in understanding rapid growth of a journal in an emerging area. J Scientometr Res.

[39] Venkatraman S, Fahd K, Kaspi S, Venkatraman R (2016). SQL Versus NoSQL movement with big data analytics. Int J Inform Technol Comput Sci.

[40] DB-Engines (2019) DB-engines ranking of graph DBMS. https://db-engines.com/en/ranking/graph+dbms. Accessed 26 May 2019

[41] Sadalage P, Fowler M (2012) NoSQL distilled: a brief guide to the emerging world of polyglot persistence. Addison-Wesley, 2012

[42] Neo4j (2019) Connections Are the Future. https://neo4j.com/product/. Accessed 9 March 2020

[43] Rossanez A, dos Reis JC (2019) Generating knowledge graphs from scientific literature of degenerative diseases. In: Abstracts of the 4th international workshop on semantics-powered data mining and analytics, Auckland, 27 October 2019

[44] Lee KW, Wu KL, Kuo HP, Yuan PL (2017). Design and validation of a knowledge map system-the case of construction industry in Taiwan. Hum Factors Ergon Manuf Serv Ind.

